# Reducing low‐dose exposure in helical TomoTherapy for locally advanced left‐sided breast cancer with a deformable image registration–based dose‐mimicking workflow

**DOI:** 10.1002/acm2.70470

**Published:** 2026-01-27

**Authors:** Chih‐Chieh Chang, Jo‐Ting Tsai, Shih‐Ming Hsu

**Affiliations:** ^1^ Department of Radiation Oncology, Shuang Ho Hospital Taipei Medical University New Taipei Taiwan, ROC; ^2^ Medical Physics and Radiation Measurements Laboratory National Yang Ming Chiao Tung University Taipei Taiwan, ROC; ^3^ Department of Biomedical Imaging and Radiological Sciences National Yang Ming Chiao Tung University Taipei Taiwan, ROC; ^4^ Graduate Institute of Clinical Medicine College of Medicine, Taipei Medical University Taipei Taiwan, ROC; ^5^ Department of Radiology, School of Medicine, College of Medicine Taipei Medical University Taipei Taiwan, ROC

**Keywords:** breast cancer radiotherapy, deformable image registration, helical TomoTherapy, radixact

## Abstract

**Background:**

Helical TomoTherapy provides highly conformal dose distributions for breast irradiation but is limited by extensive low‐dose spillage (“low‐dose bath”), contributing to increased integral dose and potential long‐term toxicities. Complete blocks can suppress low‐dose spread, but at the cost of prolonged treatment times on legacy TomoTherapy systems.

**Purpose:**

To develop and validate a deformable image registration (DIR)‐based workflow that predicts patient‐specific low‐dose distributions and generates personalized complete blocks for TomoTherapy, aiming to reduce low‐dose exposure and integral dose. A secondary objective was to determine whether Radixact, a modern helical platform, could mitigate treatment‐time penalties while preserving dosimetric benefits.

**Methods:**

Twenty‐eight patients were retrospectively analyzed (18 tangential partial‐arc volumetric modulated arc therapy [t‐VMAT], 10 TomoTherapy). DIR‐based dose prediction derived from t‐VMAT atlases was used to construct complete blocks for replanning on Hi‐Art (TOMO_RE) and Radixact (TOMO_FA). Dosimetric endpoints included target conformity, homogeneity, organ‐at‐risk (OAR) doses, and integral dose (ID). Statistical analyses used Mann–Whitney *U* test for independent cohorts and Friedman/Wilcoxon tests for paired TomoTherapy plans with Holm–Bonferroni correction.

**Results:**

TOMO_FA significantly reduced low‐dose exposure compared with TOMO_ORI, including lower contralateral lung mean dose (0.79 vs. 3.13 Gy, *p* < 0.01) and reduced Heart V5 (12.81% vs. 20.94%, *p* = 0.027). Body‐PTV ID decreased meaningfully (103.14 vs. 114.52 Gy·L, *p* = 0.012). High‐dose cardiac parameters (V25, V40) remained within clinically acceptable limits and comparable to t‐VMAT. Treatment time improved substantially on Radixact (587.2 ± 44.3 s vs. 1118.0 ± 135.5 s).

**Conclusions:**

The proposed DIR‐based complete block workflow effectively reduces low‐dose exposure and integral dose in helical TomoTherapy without compromising delivery efficiency when implemented on Radixact. TOMO_FA represents a practical, personalized planning option, particularly for patients requiring stringent low‐dose sparing.

## 1 INTRODUCTION

Breast cancer is the most frequently diagnosed cancer among women globally.^1^ Adjuvant radiotherapy is an integral component of breast cancer treatment following primary surgery that significantly improves locoregional control and increases overall survival rates.^2^ As patient survival times have lengthened, concerns about the long‐term effects of radiation exposure on normal tissues have grown stronger. Cardiac disease resulting from radiation exposure to the heart during radiotherapy for left‐sided breast cancer is a primary topic of concern. This concern is underscored by landmark studies demonstrating that the rate of major coronary events increases by 7.4% for each 1‐Gy increase in mean heart dose.^3^ Physicians now maximize dose conformity to the target volume while aggressively sparing adjacent organs at risk (OARs).

Helical TomoTherapy (TOMO) delivers intensity‐modulated radiation therapy by using a rotating gantry that enables the creation of highly conformal dose distributions. This modality exposes OARs, such as the heart and ipsilateral lung, to significantly less radiation than does conventional three‐dimensional conformal radiotherapy (3DCRT).^4^, ^5^ A drawback of helical delivery techniques is their production of a diffuse, low‐dose envelope, often termed the “low‐dose bath,” that extends over a large volume of normal tissue.^6^ This characteristic low‐dose spread inevitably increases the total body integral dose (ID). ID, defined as the total energy deposited within the body, serves as a critical parameter for evaluating the risks of long‐term radiation toxicities. Specifically, elevated ID has been correlated with an increased risk of radiation‐induced secondary malignancies (RISM) and radiation‐induced lymphopenia (RIL), both of which can adversely affect long‐term survival outcomes.^7^, ^8^ Such exposure raises substantial clinical concerns: for the contralateral breast, low‐dose exposure is linked to a long‐term risk of secondary malignancy, a concern substantiated by a large epidemiological study (WECARE) that found that women aged < 40 years who received > 1 Gy to a contralateral breast quadrant had a 2.5‐fold greater risk of developing a second primary breast cancer than did women who did not receive contralateral radiation exposure.^9^ Studies of TOMO for lung cancer have indicated that patients receiving low doses of radiation to large volumes of tissue (e.g., V_5_, percentage volume receiving ≥ 5 Gy) are at increased risk of radiation pneumonitis.^10^

The implementation of a complete block is a recognized strategy for reducing the spread of radiation to adjacent tissues during helical TOMO.^11^ By selectively limiting the gantry angles from which radiation beamlets can enter the patient, blocks can effectively curtail dose deposition in nontarget regions. The clinical effectiveness of this approach depends on the block design. Blocks should be properly defined and patient‐specific, otherwise, they may increase the amount of radiation normal tissues are exposed to by forcing fluence through less favorable beam paths.^12^ Specifically, the use of a complete block in TOMO restricts the available beam entry angles. This constraint forces the system to compensate by significantly prolonging the treatment time to achieve target coverage. Such an increase in delivery duration raises concerns regarding intrafraction motion and patient comfort, presenting a barrier to clinical implementation.

To address this challenge, recent studies have used dose prediction methods to evaluate or improve radiotherapy plans.^13^, ^14^ The present study has adapted this concept, hypothesizing that patient‐specific dose distributions can be predicted and that these predictions can be used to generate patient‐tailored, optimal complete blocks. Our primary objective was to develop and validate a workflow that minimizes low‐dose exposure to the lungs and contralateral breast in helical TOMO for female patients with locally advanced left‐sided breast cancer, a condition that is particularly difficult to treat because of the dosimetric challenges of simultaneously treating the left breast and supraclavicular fossa. This study also investigates whether integrating this workflow with a modern helical delivery platform, Radixact (Accuray Inc., Sunnyvale, CA), can simultaneously reduce treatment time while maintaining the dosimetric benefits of complete blocking. This approach suggests that the deformable image registration (DIR)‐based complete block method is broadly applicable to any delivery system capable of beam angle restriction, offering a versatile solution for optimizing plan quality.

Dose prediction methods are often based on convolutional neural networks^15^, ^16^ or DIR.^17^ We selected a DIR‐based approach for our dose prediction engine. DIR‐based prediction has several practical advantages over convolutional‐neural‐network‐based prediction. DIR requires fewer training cases, shorter training times, and fewer computational resources than convolutional neural networks.^18^, ^19^ The training library for our model was built using tangential partial‐arc volumetric modulated arc therapy (t‐VMAT) datasets. This modality was selected for its demonstrated efficacy in limiting low‐dose spread to normal tissues.^20^, ^21^

## 2 METHODS

### 2.1 Patient cohort and inclusion criteria

This retrospective, in silico study analyzed treatment plans developed for female patients with left‐sided, locally advanced breast cancer who had received postoperative radiotherapy between September 2020 and April 2024 after breast‐conserving surgery or modified radical mastectomy. The target volumes included the whole breast or chest wall (PTV_Breast) and the supraclavicular fossa (PTV_SCF). To ensure a valid comparison based on institutional practice where deep‐inspiration breath‐hold (DIBH) is not used for TOMO, treatment plans were included in this study only if the corresponding patient was treated in a free‐breathing state. The final study cohort comprised 28 clinically delivered treatment plans, which were stratified into two groups: 18 patients treated with t‐VMAT and 10 patients treated with TOMO. For the TOMO cohort, three sets of treatment plans were generated for each patient to facilitate a paired dosimetric comparison: (1) TOMO_ORI (original), the clinically delivered plan; (2) TOMO_RE (replanned), a retrospective study using the knowledge‐based complete block on the original system; and (3) TOMO_FA (fast), an optimized plan using the complete block on the Radixact system.

### 2.2 Treatment planning and dosimetry

**FIGURE 1:**
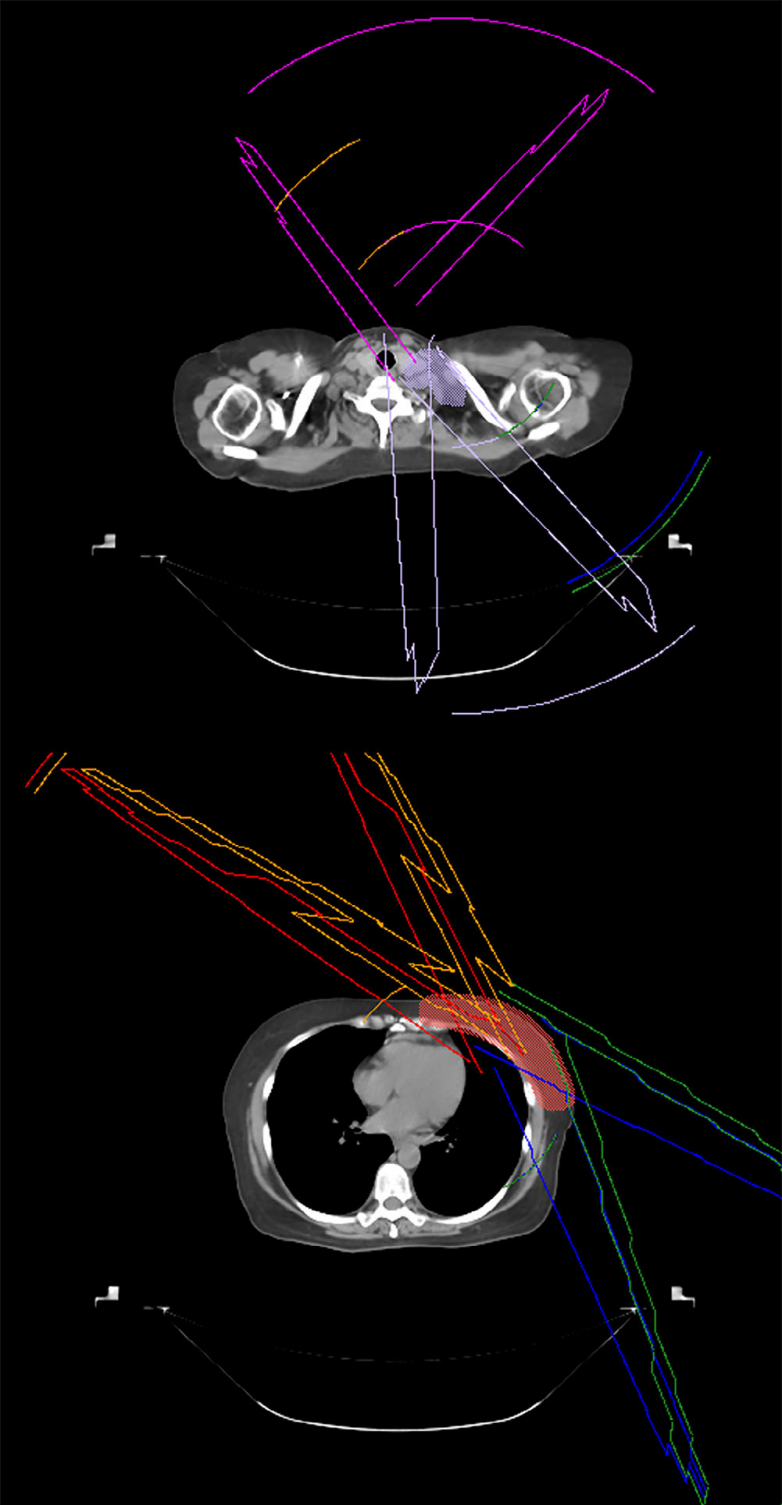
Beam arrangement of tangential VMAT. Illustration of partial‐arc beam arrangement for representative tangential partial‐arc volumetric modulated arc therapy (t‐VMAT) plan. The configuration consists of four tangential partial arcs for the breast and two complementary partial arcs for the supraclavicular fossa.

Computed tomography images, available for all plans, had been obtained using a Philips Big Bore scanner (Philips Healthcare, Amsterdam, Netherlands), with slice thickness set to 5 mm. The t‐VMAT plans, which constituted the atlas for dose prediction, had been generated using a Pinnacle^3^ (v14, Philips Radiation Oncology Systems, Fitchburg, WI) treatment planning system (TPS) and consisted of four 6‐MV tangential partial arcs to the breast and chest wall and two complementary partial arcs (a 6‐MV anterior–posterior partial arc and a 10‐MV posterior–anterior partial arc) to the supraclavicular fossa, as illustrated in Figure 1. Dose calculations had been performed with an adaptive convolution algorithm and an isotropic grid resolution of 3 mm.

The TOMO plans had been generated using Accuray planning systems. TOMO permits two types of beam‐angle restriction: direct blocking, which prohibits beamlets that traverse the block volume before reaching the target, and complete blocking, which more stringently prohibits any beamlet whose path intersects the block volume at any point. For the TOMO_ORI and TOMO_RE plans, the Accuray Hi‐Art planning system (v5.1.6) was used with a beam energy of 6 MV, a field width (FW) of 2.5 cm, fixed jaws, and a dose rate of 867 MU/min. The original clinical plans (TOMO_ORI) had employed a directional block technique, where the block structure was manually delineated based on the contralateral lung volume to reduce radiation exposure. For the TOMO_FA plans, optimization was performed on the Accuray Precision treatment planning system (v3.5) for the Radixact machine. Key parameters for TOMO_FA included a 6‐MV beam, a FW of 5.0 cm, dynamic jaws feature, and a dose rate of 1000 MU/min to enhance delivery efficiency.

### 2.3 Rationale for atlas dataset and baseline comparison

To validate the t‐VMAT cohort as a dosimetric benchmark for optimizing TOMO plans, we performed a baseline statistical comparison between the TOMO_ORI and t‐VMAT plans. This analysis was performed to verify two conditions: first, that no significant differences existed in baseline patient characteristics (e.g., height, weight, body mass index, and tumor stage); and second, that the t‐VMAT plans demonstrated superior performance in sparing normal tissues from low‐dose radiation. A dosimetric comparison was performed for target coverage metrics and dose to OARs, including the heart, lungs, contralateral breast, and esophagus.

### 2.4 Atlas‐based dose prediction

An atlas for knowledge‐based dose prediction was constructed using a DIR framework.^17^ DIR was performed with the open‐source Plastimatch toolkit (v1.9.4).^22^ Fixed and moving volumes were first aligned using a multiresolution affine stage. In this preliminary stage, a random‐search gradient optimizer with 20 iterations and 3 resolution levels (downsampling factors: 4 × 4 × 2) was used for global corrections in translation, rotation, and scaling. The resulting affine transform was then used as the input for a subsequent B‐spline deformable registration implemented natively in Plastimatch. The B‐spline registration was run over 30 iterations and at the same 3 resolution levels (4 × 4 × 2) with a uniform control‐point grid with a spacing of 50 mm in all directions. The final deformation vector field and warped moving image were exported for downstream usage.

This two‐stage registration process was applied for both atlas construction and subsequent dose prediction. For atlas construction, the computed tomography images from the 17 t‐VMAT plans were deformably registered to a randomly selected reference patient, and the corresponding 3D dose distributions were warped into the reference coordinate system using the resulting deformation vector fields. For dose prediction on a new test patient, the same procedure was used to register the test patient to the reference, generating a deformation vector field and its inverse. Volumetric similarity between the test patient and each atlas patient was quantified using a slice‐wise structural similarity index,^23^ yielding a weighting factor for each atlas case. The final predicted 3D dose for the test patient was then calculated as the weighted summation of all 18 atlas doses after they were deformed into the test patient's geometry. The entire atlas construction and dose prediction pipeline was developed and executed using custom scripts in MATLAB (R2019b; The MathWorks, Natick, MA).

### 2.5 Validation of the dose prediction model

The predictive accuracy of the DIR‐based model was evaluated using leave‐one‐out cross‐validation. The geometric accuracy of the predicted low‐dose volume was assessed using the Dice similarity coefficient (DSC) for the 2‐Gy isodose contour. The cross‐validation procedure was automated using custom MATLAB scripts. All computations were performed on a computer with an Intel Core i7‐6700 CPU, 16 GB of 2133‐MHz RAM, an Intel HD Graphics 530 GPU, and a 7200‐RPM hard disk drive.

### 2.6 TOMO replanning with a predicted complete block

**FIGURE 2:**
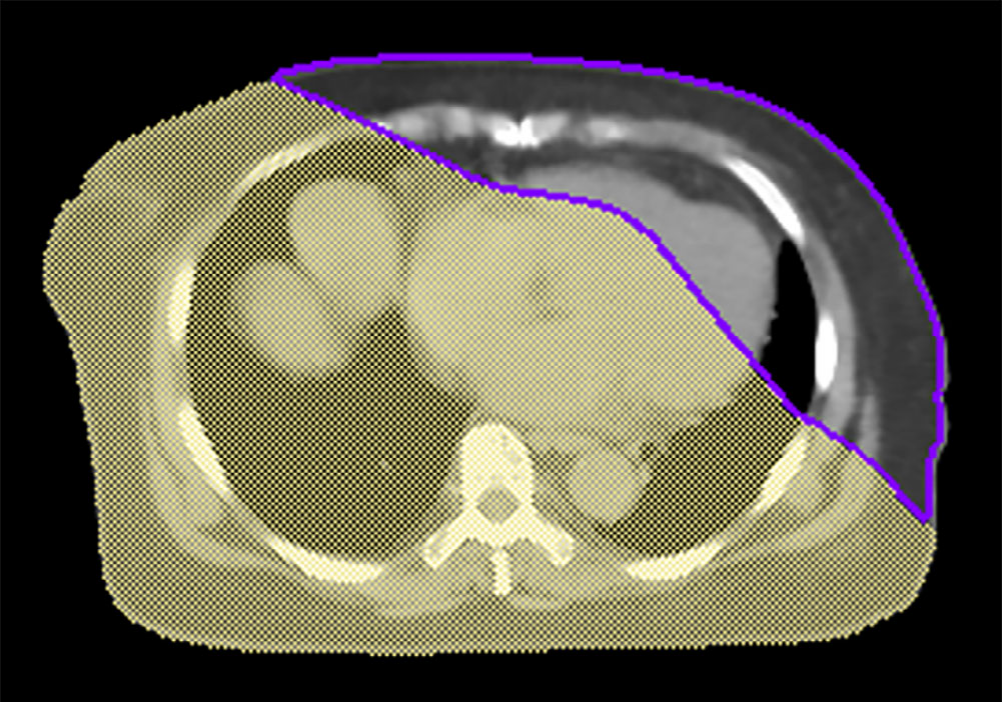
Generation of patient‐specific complete block. Generation of patient‐specific complete block. The predicted 2‐Gy isodose volume (purple contour) is subtracted from the external body contour to create the final complete block structure (yellow shaded region), which is then used to constrain the TomoTherapy plan optimization.

For each of the 10 patients in the TOMO cohort, two new plans (TOMO_RE and TOMO_FA) were generated for this in silico study to validate the proposed workflow. This process began with the generation of a patient‐specific block structure derived from the predicted 3D dose map. The predicted 3D dose map, formatted as a DICOM RTDOSE object, was imported into the Pinnacle system, where a Boolean subtraction (external body minus predicted 2‐Gy isodose volume) was performed (Figure 2). The resulting structure was smoothed to ensure geometric continuity and subsequently exported. The structure was then imported into the Accuray planning station and designated as a complete block. The optimization process was initiated using the planning constraints detailed in Table 1. For TOMO_FA planning in the Precision TPS (v3.5), the target optimization parameters differed from those used in the Hi‐Art system. Notably, the complete block (CB) only needed to be defined during the initial setup and did not require explicit dose constraints during optimization. The clinical goals for breast cases in our institution are detailed in Table 2. These replanned studies were not used for clinical treatment.

**TABLE 1:** Initial planning constraints for Hi‐Art and Precision planning system.

		Constraint value for 50 Gy (50.4 Gy)	
	Parameter	Hi‐Art	Precision
PTV_Breast	D_max_ (Gy)	< 50.00 (< 50.40)	< 52.50 (< 52.92)
	D_min_ (Gy)	>47.50 (> 47.88)	N/A
	Volume Dose (%)	V_50_ > 90 (V_50.4_ > 90)	V_51_ > 97 (V_51.4_ > 97)
PTV_SCF	D_max_ (Gy)	<50.00 (< 50.40)	< 52.50 (< 52.92)
	D_min_ (Gy)	>47.50 (> 47.88)	N/A
	Volume Dose (%)	V_50_ > 90 (V_50.4_ > 90)	V_51_ > 97 (V_51.4_ > 90)
Ipsilateral lung	D_max_ (Gy)	< 50.00	
	Volume Dose (%)	V_4_ < 35; V_8_ < 25; V_16_ < 15	
Heart	D_max_ (Gy)	<48.50 (< 48.88)	
	Volume Dose (%)	V_2.5_ < 15; V_15_ < 5	
Esophagus	D_max_ (Gy)	<36.00	
	Volume Dose (%)	V_25_ < 5	
Spinal cord	D_max_ (Gy)	<30.00	
	Volume Dose (%)	V_25_ < 5	
Contralateral breast	D_max_ (Gy)	<48.50 (< 48.88)	
	Volume Dose (%)	V_5_ < 10 (V_5_ < 15)	
Complete block	D_max_ (Gy)	<2.00	N/A
	Volume Dose (%)	V_1_ < 50	N/A

*Notes*: Values are presented for the 50 Gy plan, with values for the 50.4 Gy plan shown in parentheses where they differ. If only one value is shown, the constraint was identical for both plans.

Abbreviations: PTV, Planning target volume; SCF, Supraclavicular fossa; V_x_, percentage of the volume receiving at least x Gy

**TABLE 2:** Clinical dose–Volume goals for breast treatment planning.

Structure	Parameter	Goal
PTV	V_95%_ (%)	>95
Spinal cord	D_max_ (Gy)	<45
Heart	Mean dose (Gy)	<5
Ipsilateral lung	V_5_ (%)	<50
	V_10_ (%)	<40
	V_20_ (%)	<30
	V_30_ (%)	<20
Esophagus	D_max_ (Gy)	<40
Left ventricle	Mean dose (Gy)	<5.5

Abbreviations: PTV, planning target volume; Vx%, the proportions of the irradiation volume with doses of x% of prescribed dose to the specific target or organ volume; Vx, percentage volume receiving ≥ x Gy.

### 2.7 Integral dose calculation

To evaluate the total energy deposited in the patient, the ID was calculated for the heart, ipsilateral lung, and the whole body minus the target volumes (Body‐PTV). ID was defined as the product of the mean dose and the volume of the structure, assuming a tissue density of 1 g/cm^3^ for all soft tissues. The unit of ID was expressed in Gray·Liter (Gy·L).

### 2.8 Statistical analysis

All dosimetric parameters were analyzed in Computational Environment for Radiological Research software, a platform based on MATLAB.^24^ Subsequent statistical evaluations were performed using IBM SPSS Statistics (v29.0), with *p* < 0.05 indicating statistical significance. Given the cohort size, non‐parametric tests were utilized. Fisher's exact test was used to compare categorical variables between independent groups. The Mann–Whitney *U* test was applied for comparisons of continuous variables between the TOMO cohort and the independent t‐VMAT cohort. For comparisons within the TOMO cohort (n = 10), where TOMO_ORI, TOMO_RE, and TOMO_FA plans were generated for the same patients, the Friedman test was employed to detect overall differences. Significant results were followed by post‐hoc pairwise comparisons using the Wilcoxon signed‐rank test. Holm‐Bonferroni correction was applied manually to control family‐wise error. The analysis was structured around three key comparisons: TOMO_FA versus t‐VMAT, TOMO_FA versus TOMO_ORI, and TOMO_FA versus TOMO_RE.

### 2.9 Use of artificial intelligence

During the preparation of this manuscript, the authors utilized the generative AI tools Gemini (Google) and ChatGPT (OpenAI) to enhance language, grammar, and overall readability. In line with COPE and Wiley best practices, these tools were used solely for linguistic support and did not contribute to the generation, analysis, or interpretation of scientific data. The authors retain full responsibility for all scientific content and conclusions presented herein.

## 3 RESULTS

### 3.1 Patient and treatment characteristics

A total of 28 patients were included in this study, with 18 in the t‐VMAT cohort and 10 in the TOMO cohort. The baseline characteristics for both groups are detailed in Table 3. No significant differences (*p *> 0.05) were noted in most clinical and anatomical characteristics (e.g., age, body mass index, organ volumes) between the cohorts, confirming that they were well‐matched. A significant difference was observed in PTV_SCF volume (*p *= 0.049).

**TABLE 3:** Patient and treatment characteristics for t‐VMAT and TOMO cohorts.

	t‐VMAT (*n* = 18)	TOMO (*n* = 10)	*p*‐value
Age (years)	57.83 ± 12.17	58.00 ± 10.71	1.000
Height (cm)	156.22 ± 6.67	153.45 ± 6.80	0.524
Weight (kg)	59.56 ± 11.50	61.19 ± 7.71	0.464
BMI (kg/m^2^)	24.24 ± 3.29	26.03 ± 3.14	0.099
PTV_Breast (cc)	638.63 ± 201.47	615.59 ± 212.44	0.701
PTV_SCF (cc)	92.88 ± 13.89	80.32 ± 18.79	0.049
Heart (cc)	438.58 ± 97.74	507.37 ± 103.24	0.061
Ipsilateral lung (cc)	1135.04 ± 409.50	958.95 ± 206.91	0.093
Contralateral lung (cc)	1455.31 ± 502.12	1238.75 ± 219.42	0.314
Contralateral breast (cc)	511.28 ± 116.30	484.44 ± 141.67	0.962
Surgery type			1.000
BCS	10 (55.56%)	5 (50%)	
MRM	8 (44.44%)	5 (50%)	
Prescribed dose			1.000
50.00 Gy	11 (61.11%)	6 (60%)	
50.40 Gy	7 (38.89%)	4 (40%)	
Tumor stage^*^			0.634
T0 ‐ T2	15 (83.33%)	7 (70%)	
T3 ‐ T4	3 (16.67%)	3 (30%)	
Nodal stage^*^			0.601
N0 ‐ N2	16 (88.89%)	8 (80%)	
N3 ‐ N4	2 (11.11%)	2 (20%)	

*Note*: Data are presented as mean ± standard deviation or *n* (%).

Abbreviations: BCS, Breast‐conserving surgery; BMI, Body mass index; MRM, Modified radical mastectomy; PTV, Planning target volume; SCF, Supraclavicular fossa.

* Pathologic stage.

### 3.2 Dosimetric comparison and rationale for block design

**FIGURE 3:**
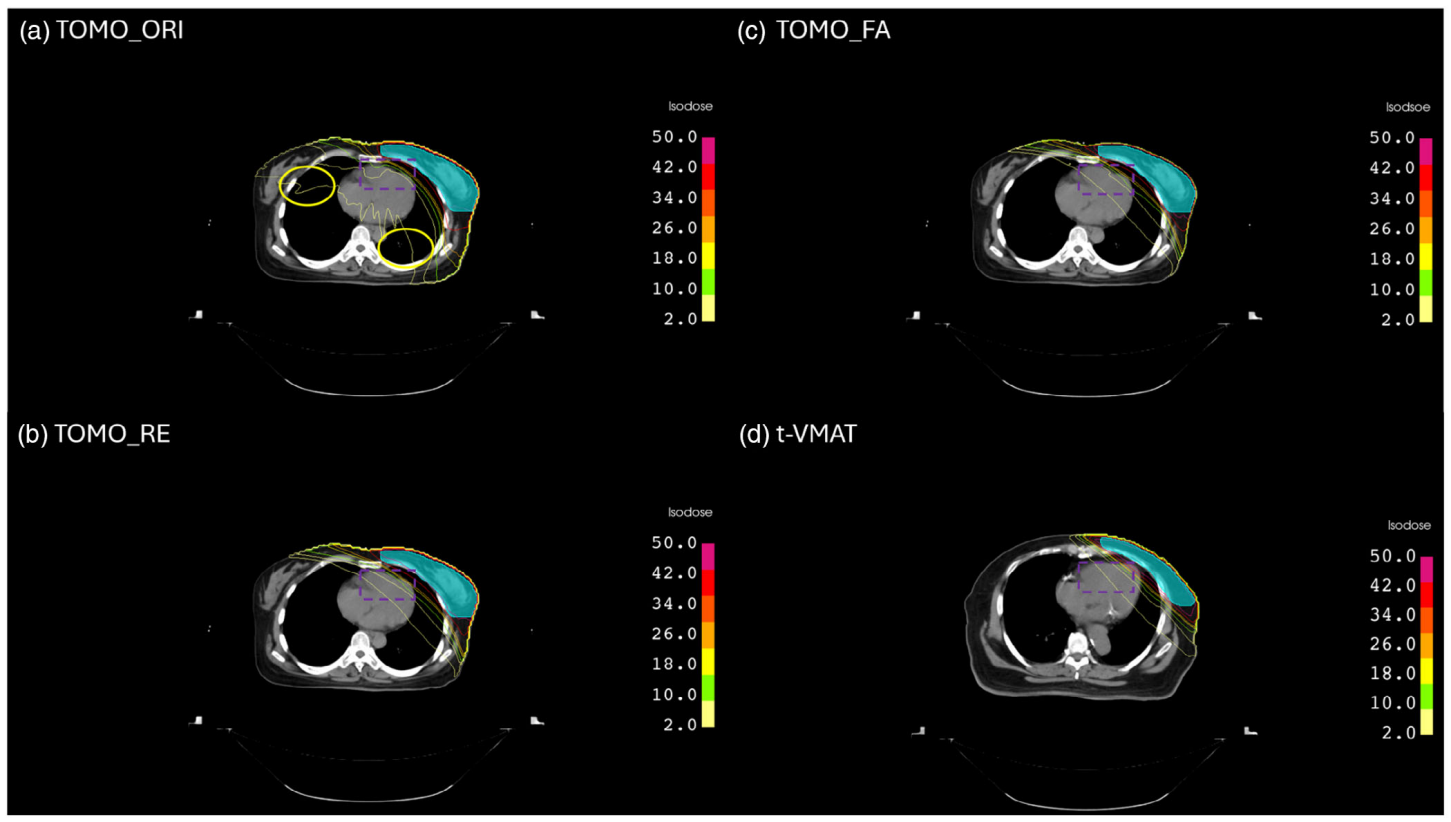
Comparative axial dose distributions. Comparative axial dose distributions for a representative left‐sided breast cancer case. (a) The original TomoTherapy plan (TOMO_ORI) demonstrates a wide low‐dose spread to both lungs and the contralateral breast (yellow circles) but superior high‐dose conformity around the heart (purple dashed box), resulting in lower V40 and V25. (b) The replanned TomoTherapy plan with the knowledge‐based complete block (TOMO_RE) markedly reduces the low‐dose bath. (c) The fast‐approach Radixact implementation of the same complete‐block workflow (TOMO_FA) preserves low‐dose sparing and target conformity while achieving a clinically acceptable treatment time. (d) The tangential partial‐arc VMAT plan (t‐VMAT) from an anatomically similar patient serves as the benchmark for conventional low‐dose sparing.

The initial dosimetric comparison between the t‐VMAT and TOMO_ORI plans confirmed a clear trade‐off, providing the rationale for our block design strategy. TOMO_ORI plans demonstrated superior dose conformity index (CI) for both the breast (CI_Breast: 0.90 vs 0.88, *p* = 0.049) and the supraclavicular fossa (CI_SCF: 0.95 vs 0.88, *p* = 0.014), as well as superior dose homogeneity index (HI) for the supraclavicular fossa (HI_SCF: 0.09 vs 0.15, *p* = 0.017). However, this conformity came at the cost of significantly higher low‐dose spillage. Specifically, TOMO_ORI resulted in a larger low‐dose cardiac volume (Heart V5: 20.94% vs 10.82%, *p* < 0.001) and higher mean doses to the contralateral lung (3.13 Gy vs 0.75 Gy, *p* < 0.001) and breast (5.64 Gy vs 2.71 Gy, *p* = 0.001) compared to t‐VMAT (Figure 3). This baseline difference justified using the low‐dose region from t‐VMAT plans as the template for designing patient‐specific complete blocks.

The impact of integrating these blocks into the TOMO plans (TOMO_RE and TOMO_FA) is detailed in Tables 4, 5, 6. Both blocked techniques successfully translated the low‐dose advantages of t‐VMAT into the TOMO platform through the complete block (CB) technique. Compared to TOMO_ORI, both TOMO_RE, and TOMO_FA reduced the Heart V5 (to 15.83% and 12.81%, respectively). However, this reduction reached statistical significance only in the TOMO_FA plans (*p* = 0.027), while the reduction in TOMO_RE approached but did not reach significance (*p* = 0.064). Both techniques significantly reduced the contralateral lung mean dose (to 0.72 Gy and 0.79 Gy; *p* < 0.01), effectively mitigating the low‐dose bath (Figure 3).

**TABLE 4:** Targets dose and treatment time comparison among tangential VMAT, original TomoTherapy, replanned TomoTherapy, and Radixact plans.

Parameter	t‐VMAT (*n* = 18, Mean ± SD)	TOMO_ORI (*n* = 10, Mean ± SD)	TOMO_RE (*n* = 10, Mean ± SD)	TOMO_FA (*n* = 10, Mean ± SD)	*p*_1_ (FA vs t‐VMAT)	*p*_2_ (FA vs ORI)	*p*_3_ (FA vs RE)
PTV_Breast V95% (%)	96.39 ± 2.04	97.03 ± 0.96	96.27 ± 0.95	96.33 ± 1.21	0.517	0.551	0.953
CI_Breast	0.88 ± 0.03	0.90 ± 0.03	0.91 ± 0.01	0.92 ± 0.02	0.002	0.413	0.324
HI_Breast	0.19 ± 0.11	0.14 ± 0.04	0.18 ± 0.05	0.20 ± 0.06	0.374	0.239	0.292
PTV_SCF V95% (%)	96.61 ± 1.82	98.37 ± 2.00	98.47 ± 2.16	97.65 ± 1.66	0.119	0.750	0.316
CI_SCF	0.88 ± 0.05	0.95 ± 0.07	0.96 ± 0.04	0.95 ± 0.02	< 0.001	1.000	1.000
HI_SCF	0.15 ± 0.05	0.09 ± 0.07	0.11 ± 0.07	0.13 ± 0.09	0.229	0.464	0.465
Treatment time (s)	—	821.8 ± 92.6	1118.0 ± 135.5	587.2 ± 44.3	—	< 0.001	< 0.001

Abbreviations: CI, conformity index; HI, homogeneity index; PTV, planning target volume; SCF, supraclavicular fossa; SD, standard deviation; TOMO_FA, Radixact with dynamic jaws plan; TOMO_ORI, original TomoTherapy plan; TOMO_RE, replanned TomoTherapy plan; t‐VMAT, tangential volumetric modulated arc therapy; Vx%, the proportion of the structure volume receiving x% of the prescribed dose.

**TABLE 5:** Critical organ dose comparison among tangential VMAT, original TomoTherapy, replanned TomoTherapy, and Radixact plans.

Parameter	t‐VMAT (*n* = 18, Mean ± SD)	TOMO_ORI (*n* = 10, Mean ± SD)	TOMO_RE (*n* = 10, Mean ± SD)	TOMO_FA (*n* = 10, Mean ± SD)	*p*_1_ (FA vs t‐VMAT)	*p*_2_ (FA vs ORI)	*p*_3_ (FA vs RE)
Heart mean dose (Gy)	3.41 ± 0.83	4.48 ± 0.59	4.15 ± 1.10	3.66 ± 1.13	0.326	0.098	0.041
Heart V40 (%)	1.05 ± 0.76	0.13 ± 0.19	0.71 ± 0.58	0.50 ± 0.41	0.061	0.030	0.075
Heart V25 (%)	2.80 ± 1.37	1.50 ± 1.03	3.84 ± 1.96	2.91 ± 1.93	0.666	0.020	0.020
Heart V5 (%)	10.82 ± 3.94	20.94 ± 4.69	15.83 ± 5.06	12.81 ± 5.61	0.240	0.027	0.006
Heart V2 (%)	35.87 ± 8.71	81.33 ± 15.43	38.76 ± 7.25	38.40 ± 9.02	0.221	0.006	0.695
Ipsilateral lung mean dose (Gy)	11.46 ± 2.06	12.83 ± 1.50	11.92 ± 1.61	11.84 ± 1.32	0.829	0.393	0.846
Ipsilateral lung V20 (%)	22.09 ± 4.97	24.89 ± 3.81	23.24 ± 3.38	23.35 ± 3.18	0.649	0.645	0.770
Ipsilateral lung V10 (%)	30.82 ± 5.94	36.54 ± 4.22	34.06 ± 4.62	34.71 ± 3.09	0.080	0.645	0.645
Ipsilateral lung V5 (%)	41.44 ± 6.08	49.06 ± 3.68	44.51 ± 4.35	45.98 ± 4.38	0.108	0.168	0.168
Contralateral lung mean dose (Gy)	0.75 ± 0.28	3.13 ± 0.84	0.72 ± 0.10	0.79 ± 0.11	0.089	0.006	0.014
Contralateral breast mean dose (Gy)	2.71 ± 2.29	5.64 ± 2.00	3.58 ± 0.67	4.13 ± 1.23	0.006	0.211	0.211
Esophagus Dmax (Gy)	36.92 ± 2.11	35.43 ± 3.76	35.95 ± 3.97	37.04 ± 3.04	0.810	0.193	0.863

Abbreviations: SD, standard deviation; TOMO_FA, Radixact with dynamic jaws plan; TOMO_ORI, original TomoTherapy plan; TOMO_RE, replanned TomoTherapy plan; t‐VMAT, tangential volumetric modulated arc therapy; Vx, percentage volume receiving ≥ x Gy.

**TABLE 6:** Integral dose comparison among tangential VMAT, original TomoTherapy, replanned TomoTherapy, and Radixact plans.

Parameter	t‐VMAT (*n* = 18, Mean ± SD)	TOMO_ORI (*n* = 10, Mean ± SD)	TOMO_RE (*n* = 10, Mean ± SD)	TOMO_FA (*n* = 10, Mean ± SD)	*p*_1_ (FA vs t‐VMAT)	*p*_2_ (FA vs ORI)	*p*_3_ (FA vs RE)
Heart integral dose (Gy·L)	1.51 ± 0.64	2.26 ± 0.54	2.09 ± 0.63	1.85 ± 0.68	0.144	0.074	0.029
Ipsilateral lung integral dose (Gy·L)	12.74 ± 3.74	12.23 ± 2.53	11.25 ± 1.73	11.24 ± 2.03	0.303	0.387	1.000
Body–PTV integral dose (Gy·L)	82.35 ± 15.20	114.52 ± 20.96	97.43 ± 17.53	103.14 ± 20.48	0.012	0.012	0.012

Abbreviations: SD, standard deviation; TOMO_FA, Radixact with dynamic jaws plan; TOMO_ORI, original TomoTherapy plan; TOMO_RE, replanned TomoTherapy plan; t‐VMAT, tangential volumetric modulated arc therapy.

However, the delivery system played a decisive role in the overall plan quality and efficiency. While TOMO_RE successfully reduced low doses, it did so at the expense of a significantly prolonged treatment time (1118.0 ± 135.5 s) and increased high‐dose cardiac exposure (Heart V25 increased to 3.84%). In contrast, the optimized TOMO_FA technique not only maintained the low‐dose sparing benefits but also drastically reduced the treatment time to 587.2 ± 44.3 s (*p* < 0.001 vs. TOMO_ORI and TOMO_RE). Furthermore, TOMO_FA controlled the high‐dose trade‐off more effectively. Although the Heart V25 in TOMO_FA (2.91 ± 1.93%) was statistically higher than in TOMO_ORI (1.50 ± 1.03%, *p* = 0.020), it was significantly lower than in TOMO_RE (3.84 ± 1.96%, *p* = 0.020) and statistically indistinguishable from the t‐VMAT benchmark (2.80 ± 1.37%, *p* = 0.666), suggesting no excess high‐dose risk relative to standard clinical practice in our department. Regarding the very high‐dose region, although the Heart V40 in TOMO_FA (0.50 ± 0.41%) was statistically higher than in TOMO_ORI (0.13 ± 0.19%, *p* = 0.030), the absolute volume remained very low (< 1%) and was substantially lower than in the VMAT group (1.05 ± 0.76%).

#### 3.2.1 Integral dose analysis

Consistent with the reduction in low‐dose spillage, TOMO_FA demonstrated a favorable ID profile (Table 6). The Body‐PTV ID in TOMO_FA (103.14 ± 20.48 Gy·L) was significantly lower than in the TOMO_ORI plans (114.52 ± 20.96 Gy·L, *p* = 0.012). Regarding the ipsilateral lung, although the ID was lower in TOMO_FA (11.24 ± 2.03 Gy·L) compared to TOMO_ORI (12.23 ± 2.52 Gy·L), the difference did not reach statistical significance (*p* > 0.05). Notably, contrary to concerns regarding helical delivery, the Heart ID in TOMO_FA (1.85 ± 0.68 Gy·L) was statistically comparable to that of the t‐VMAT group (1.51 ± 0.64 Gy·L, *p* = 0.144).

#### 3.2.2 Target coverage and conformity

All three TOMO techniques maintained superior target conformity compared to t‐VMAT. TOMO_FA achieved a CI_Breast of 0.92 ± 0.02 and CI_SCF of 0.95 ± 0.02, both significantly higher than t‐VMAT (0.88 ± 0.03 and 0.88 ± 0.05, respectively; *p* < 0.01). Target coverage (V95%) and homogeneity (HI) in TOMO_FA were not statistically different from TOMO_ORI or t‐VMAT (*p* > 0.05), indicating that the use of complete blocks did not compromise target quality.

### 3.3 Dose prediction model validation

**FIGURE 4:**
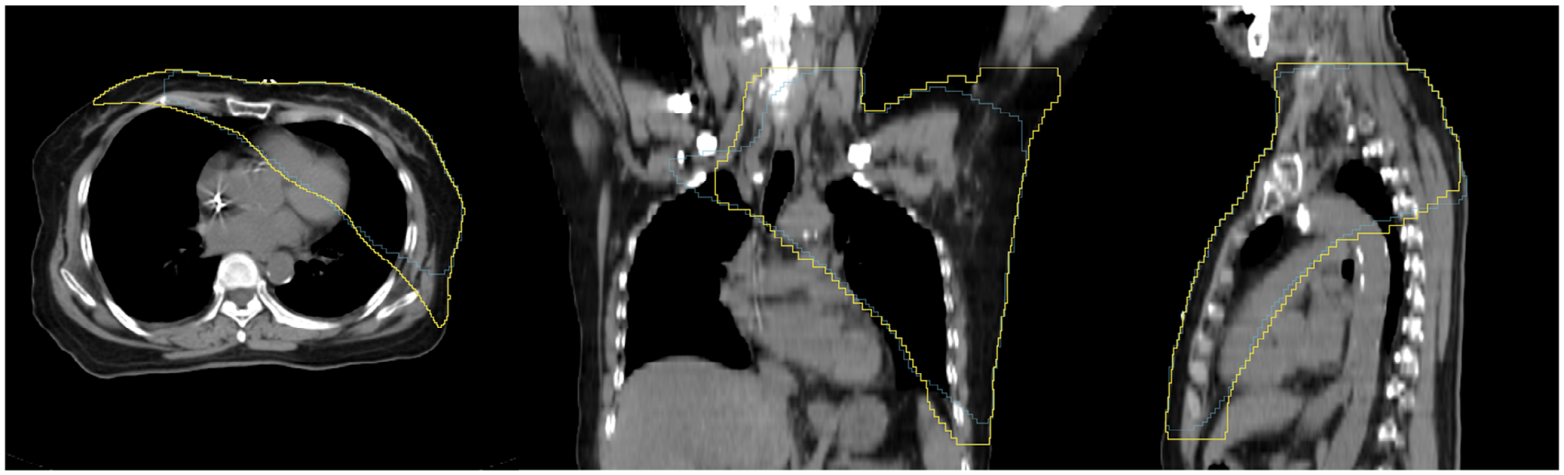
Validation of dose prediction model. Multi‐planar view of leave‐one‐out cross‐validation results for predicted 2‐Gy isodose volume. The predicted volume (yellow contour) shows high spatial concordance with the actual volume (blue contour) in the (a) axial, (b) coronal, and (c) sagittal views. The mean dice similarity coefficient across cases was 0.858 ± 0.02, confirming the accuracy of the deformable image registration (DIR)‐based dose prediction.

The dose prediction model, developed using DIR, demonstrated robust performance in leave‐one‐out cross‐validation. The mean DSC for the dose region exceeding 2 Gy was 0.858 ± 0.02, indicating a high degree of spatial concordance between predicted and actual low‐dose distributions, as shown in the multiplanar comparison in Figure 4. The model also exhibited computational efficiency, with an average prediction time of 50.63 ± 8.94 s per case.

## 4 DISCUSSION

The principal contribution of this investigation is the validation of a novel clinical workflow that uses DIR‐based dose‐mimicking of t‐VMAT plans to generate patient‐specific complete blocks for TOMO. The results demonstrate that this methodology is not only technically feasible but also highly effective, achieving a significant reduction in the characteristic low‐dose spillage associated with TOMO while preserving the modality's inherent advantages in target dose conformity and homogeneity. Crucially, the integration of this workflow with the Radixact system enabled the delivery of these highly modulated plans within a clinically efficient timeframe.

Although knowledge‐based planning and dose‐mimicking techniques have been applied to plan evaluation and optimization across various radiotherapy domains, the present study is the first to adapt this concept for the explicit construction of a complete block within the TOMO framework. The selection of DIR as the prediction engine was predicated on its practical advantages for clinical settings with limited resources; specifically, DIR demands less specialized computational hardware and a smaller training dataset relative to deep‐learning alternatives such as convolutional neural networks. A limitation of DIR is that its accuracy can be compromised in regions with steep dose gradients, such as the interface between the PTV and adjacent OARs, which can lead to a “blurring” effect that may misrepresent the predicted dose distribution. This study's aim was to predict the general shape of the low‐dose bath characterized by a relatively flat dose distribution; therefore, this limitation is substantially less pronounced.^13^, ^25^ The high geometric accuracy of our model, substantiated by a mean DSC of 0.858 for the 2‐Gy isodose volume, demonstrates that DIR is a robust and efficient tool for this particular application. Dose prediction in high‐gradient regions remains a key area of investigation for adaptive radiotherapy and dose accumulation.^26^

The translation of this blocking strategy into clinical practice, however, requires a careful balance between low‐dose sparing, high‐dose risk, and delivery efficiency. Our initial attempt with TOMO_RE on the legacy system revealed the mechanical challenges of applying complete blocks: the restriction of beam entry angles forced the optimizer to significantly prolong treatment time (> 18 min) and redistribute dose into high‐dose cardiac regions (Heart V25: 3.84%). In contrast, the TOMO_FA workflow on the Radixact system effectively resolved the efficiency concern. Leveraging the dynamic jaws feature and higher dose rate, TOMO_FA reduced the treatment time to under 10 min.^27^ Regarding the high‐dose trade‐off, while TOMO_FA exhibited a Heart V25 (2.91%) higher than the original plan, it was statistically indistinguishable from the t‐VMAT benchmark (2.80%, *p* = 0.666). Similarly, although the Heart V40 in TOMO_FA (0.50%) was statistically higher than in TOMO_ORI (0.13%), this absolute volume is clinically negligible (< 1%) and remains substantially lower than that of standard t‐VMAT plans (1.05%). This suggests that the high‐dose spillover in our optimized workflow is well within the safety margins of current clinical practice.

While TOMO_FA demonstrates improved efficiency for conventional fractionation (approximately 2.0 Gy per fraction), contemporary guidelines and randomized trials support moderately hypofractionated whole‐breast and regional nodal irradiation regimens (e.g., 40 Gy in 15 fractions of 2.67 Gy) as a standard of care in many settings.^28^, ^29^ Implementing such hypofractionated regimens with helical delivery would, in principle, require a proportional increase in monitor units per fraction, and is therefore expected to extend beam‐on time by approximately 30% compared with the conventionally fractionated TOMO_FA plans analyzed herein, assuming similar modulation and dose‐rate settings. Consequently, despite the efficiency gains of the Radixact system, prolonged immobilization remains a limitation relative to VMAT. Patient selection is therefore critical; this workflow is best suited for compliant patients with high postural stability, verified by daily image‐guided radiotherapy (IGRT) or surface‐guided radiotherapy (SGRT), to minimize clinical uncertainty associated with intrafraction motion.

A theoretical limitation often cited in radiotherapy planning is that optimization merely redistributes dose without significantly reducing the total energy delivered.^30^, ^31^ However, our results indicate a divergence from this assumption in the context of helical delivery with complete blocking. We observed a significant reduction in the Body‐PTV ID with TOMO_FA compared to TOMO_ORI (*p* = 0.012). This net reduction in energy deposition is primarily driven by the drastic suppression of the extensive low‐dose bath. Our data show that the complete block technique effectively shielded a substantial volume of tissue from very low‐dose exposure (e.g., Heart V2 reduced from 81% to 38%). Although there was a compensatory increase in high‐dose volumes (e.g., V25), the volumetric reduction in the low‐dose region was substantial enough to decrease the total ID. This finding aligns with previous studies, which reported that technique‐specific optimization could influence ID.^32^

The observed reduction in ID and low‐dose spillage in TOMO_FA likely reflects the synergistic effect of the patient‐specific complete block and the dynamic jaws feature. The dynamic jaws feature makes the use of a wide 5.0 cm field width clinically acceptable for improving delivery efficiency, while at the same time mitigating the longitudinal penumbra that would otherwise accompany such wide fields. Unlike fixed jaws, dynamic jaws feature adapts the beam aperture to the cranial–caudal target boundaries, effectively reducing the extent of the longitudinal penumbra. This mechanism is consistent with the lower total energy deposited in the patient observed in the TOMO_FA cohort compared with TOMO_ORI, as well as with prior report demonstrating reduced penumbra, integral dose, and treatment time when dynamic jaws feature is employed.^33^

The clinical relevance of these dosimetric shifts is further contextualized by established radiobiological models. The observed reduction in mean contralateral breast dose is particularly salient, given that the WECARE study found that women aged younger than 40 years who received > 1 Gy to a contralateral quadrant experienced a 2.5‐fold higher incidence of second primary malignancies. In parallel, the documented increase in the heart V25 and V40 parameters necessitates careful evaluation. According to QUANTEC guidelines, keeping the heart V25 below 10% is recommended to limit the risk of long‐term cardiac mortality.^34^ Although the heart V25 in the TOMO_FA plans (2.91%) remained well below this threshold, its increase from the baseline warrants clinical vigilance, particularly for patients at risk of pericardial disease, which is associated with larger high‐dose volumes.^35^

Consequently, for younger female patients for whom the minimization of lifetime risk for secondary contralateral breast cancer is a primary objective, t‐VMAT might represent the more prudent therapeutic choice. Conversely, for patients presenting with complex target anatomy where exceptional dose conformity is critical, the TOMO_FA plan arguably provides a more advantageous balance between target coverage and organ‐at‐risk sparing. Ultimately, treatment decisions should consider patient anatomy, comorbidities, and long‐term risk profiles.

It is important to emphasize that the DIR‐based dose‐mimicking workflow proposed in this study is not exclusively limited to the Radixact system. The core principle—utilizing patient‐specific dose prediction to define optimal avoidance sectors—is a universal methodology applicable to any radiotherapy delivery system or treatment planning system (TPS) that supports beam blocking or sector avoidance functions. As long as the delivery platform offers sufficient flexibility to restrict beam entry angles, this personalized blocking strategy can be implemented to effectively mitigate low‐dose spillage, providing a versatile tool for plan optimization across different radiotherapy modalities.

This study has several limitations. First, this is a proof‐of‐concept investigation aimed at validating the feasibility and efficacy of a novel workflow rather than a large‐scale analysis of clinical outcomes. Thus, the modest sample size, particularly of the TOMO cohort (*n* = 10), restricts the generalizability of the dosimetric findings. Second, the retrospective nature of the study and the significant difference identified in the PTV_SCF volume (*p* = 0.049) entail a high likelihood of confounding.

Third, we acknowledge that this study was conducted under free‐breathing conditions, whereas DIBH is widely regarded as a standard motion management technique for left‐sided breast cancer to reduce heart dose and intra‐fraction motion. Although the Radixact system has integrated the VitalHold platform (developed with C‐RAD AB, Uppsala, Sweden) to support surface‐guided DIBH, this functionality is currently available only for TomoDirect delivery^36^ and had not been implemented on legacy TomoTherapy systems during the period of patient accrual in this study. As helical delivery was used exclusively in our planning and delivery, DIBH was not feasible in this clinical context.

Additionally, regarding specific dosimetric endpoints, this study did not distinguish between cardiac substructures, such as the left anterior descending artery, which are known to be critical predictors of specific cardiac toxicities.^37^ Finally, the scope of this study extended only to a dosimetric analysis. Long‐term clinical data pertaining to toxicity and patient outcomes are required to establish the potential therapeutic benefit of the proposed technique.

## 5 CONCLUSION

This study successfully validated a novel, time‐efficient workflow for generating patient‐specific complete blocks in TOMO using a DIR‐based dose‐mimicking technique. By integrating this workflow with the modern Radixact system, we demonstrated that the significant reduction in low‐dose spillage achieved by complete blocking does not necessitate a compromise in treatment efficiency. The optimized TOMO_FA technique reduced the delivery time to under 10 min. Critically, the significant reduction in the body ID offers a potential long‐term benefit in reducing secondary malignancy risks. This optimized workflow represents a clinically viable and personalized treatment option, particularly for younger breast cancer patients who require stringent low‐dose protection. This workflow should not be regarded as a universally superior technique, but rather as an additional clinical tool that enables more tailored treatment planning.

## AUTHOR CONTRIBUTION

Chih‐Chieh Chang designed the study, developed the methodology and software pipeline, performed the data analysis, and wrote the original manuscript. Jo‐Ting Tsai provided clinical interpretation of the dosimetric results. Shih‐Ming Hsu supervised the work and provided critical revisions to the manuscript. All authors have read and approved the final manuscript.

## TRIAL REGISTRATION

This study was approved by the Joint Institutional Review Board of Taipei Medical University, Taiwan (N202412025).

## CONFLICT OF INTEREST STATEMENT

The authors declare no conflicts of interest.

## Data Availability

The datasets generated and/or analyzed during the current study are not publicly available due to patient privacy and ethical restrictions but are available from the corresponding author on reasonable request.
